# The effects of cross-language overlap and semantic transparency on the processing of L2 collocations

**DOI:** 10.3389/fpsyg.2024.1417786

**Published:** 2024-08-29

**Authors:** Abdulaziz Altamimi

**Affiliations:** College of Languages and Translation, Imam Mohammad Ibn Saud Islamic University (IMISU), Riyadh, Saudi Arabia

**Keywords:** language processing, collocation processing, semantic transparency, congruency, L2 proficiency

## Abstract

Although extensive research has been carried out on collocation processing, it is still unclear how cross-language overlap and transparency influence the processing of collocations by L2 learners. In the current study, a phrase judgment task was used to investigate the processing of congruent (i.e., exist in both English and Arabic) and incongruent collocations (i.e., exist only in English) by Arabic non-native speakers of English. The semantic transparency of the items was controlled for. Results demonstrated the effect of congruency on processing: congruent items yielded more correct responses and faster response times than incongruent items. The effect of congruency was modulated by proficiency, with congruency having a stronger effect on lower-proficiency learners than higher-proficiency learners. Transparency had no effect, with no differences in response times and accuracy between transparent and opaque collocations. The findings have implications for the learning and teaching of L2 collocations.

## Introduction

Recently, exploring collocation learning and processing has attracted increasing research attention. From a frequency-based approach, collocations can be defined as two or more words that co-occur in close proximity more frequently than would be expected by chance alone (e.g., strong wind; Carter, 1988; Hoey, 1991; Sinclair, 1991; Moon, 1998). Collocations are ubiquitous in language use, accounting for “70% of everything we say, hear, read, or write” (Hill, 2000, p. 53). This means that it is essential for second language learners to master collocational knowledge to achieve native-like competence in the second language (L2; Siyanova-Chanturia and Pellicer-Sánchez, 2019) and to develop effective communication in the L2 (Hill, 2000).

Despite the importance and ubiquity of collocations in language use, research shows that L2 collocations are difficult to acquire for L2 learners (Durrant and Schmitt, 2010; Li and Schmitt, 2010; Laufer and Waldman, 2011). L2 learners seem to produce a large number of non-collocate/non-native-like word combinations even at advanced levels (e.g., ^*^do a mistake; Gabrys-Biskup, 1992; Bahns and Eldaw, 1993; Granger, 1998; Foster, 2001; Nesselhauf, 2003; Boers et al., 2014). It appears that L2 learners overuse non-standard forms of collocations while they underuse frequently used collocations (Granger, 1998; Foster, 2001). Crucially, about one-third of L2 learners' collocations are unacceptable (e.g., reach an aim; Nesselhauf, 2005). In Nesselhauf's (2005) study, L1 influence accounted for almost half of the collocation errors that learners made. Further, Peters (2016) showed that non-congruency (i.e., when the collocations have no equivalent in the L2) impedes collocation learning.

Some collocations are congruent, meaning they have literal translation equivalents in two languages (e.g., the phrase *play a role* is an acceptable collocation with the same meaning in both English and Arabic). Other collocations are incongruent as they exist in only one language (i.e., the English collocation *catch a cold* does not have a direct translation equivalent with the same meaning in Arabic). Previous research has demonstrated the effects of L1-L2 collocational congruency on collocation processing (Yamashita and Jiang, 2010; Wolter and Gyllstad, 2011, 2013; Wolter and Yamashita, 2017). These studies showed that collocations are processed faster when the form and meaning of collocations are shared in the two languages. A recent study by Boone et al. (2023) demonstrated an effect of congruency and proficiency on the learning of L2 collocations. However, although previous research has explored congruency effects, little is known about the combined effects of congruency, L2 proficiency, and semantic transparency on the processing of L2 collocations. This paper aims to explore the combined effects of congruency, L2 proficiency, and semantic transparency on the processing of L2 collocations. Examining factors that influence L2 collocation processing should help better understand collocation learning and thus inform the teaching of L2 collocations.

## Background

### The role of congruency in collocation processing

The two languages of bilinguals are thought to interact dynamically with each other (De Groot, 2011) due to the parallel activation of the languages when using one (Bialystok, 2011). This is supported by Kroll and Stewart's (1994) revised hierarchical model (RHM), which assumes that the early stages of L2 learning involve co-activation of the two languages, where the L1 is used to mediate access to the L2. The co-activation of languages can be explained by an automatic spread of activation to similar items, which is blind to language identification (Dijkstra and Van Heuven, 2002). This non-selective view of processing accounts for congruency effects when collocations are shared in the two languages.

Adult L2 learners are different from monolingual children learning L1 in that they come to L2 with a cognitive system that has already been wired with previously encoded L1 patterns (MacWhinney, 2017). This implies that L2 learners have a repertoire of formulaic sequences, including collocations, already stored in their mental lexicons (Conklin and Carrol, 2019). Yamashita and Jiang (2010) and Carrol et al. (2016) suggest that L2 learners bring their pre-existing knowledge of L1 patterns to the task of language learning, which may interfere with any new non-corresponding L2 patterns. According to Conklin and Carrol (2019), “acquiring new forms in the L2 is an effortful process that requires high levels of exposure, not simply to instantiate new patterns, but in some cases to reconfigure the way the same ideas are expressed” (p. 65). An example is the English collocation *take a photo* and its German collocation counterpart *ein Foto machen*, which literally translates to “make a photo.” German speakers learning English as an L2 may incorrectly say *make a photo* in English under the influence of their previously encoded German collocation (Gyllstad, 2005). Thus, it is likely that L2 learners produce malformed L2 collocations as a result of translating L1 collocations that are not matched in the L2. In contrast, when L1 collocations are matched in the L2, it could facilitate processing (Yamashita and Jiang, 2010; Wolter and Gyllstad, 2013).

Several studies have explored the effect of cross-language overlap on collocation processing (e.g., Yamashita and Jiang, 2010; Wolter and Gyllstad, 2011, 2013; Pritchett et al., 2016; Ding and Reynolds, 2019; Sonbul and El-Dakhs, 2020; Fang and Zhang, 2021). Previous studies have often assessed the role of congruency using a phrasal judgment task, in which participants judge whether a word combination is commonly used in English or not (e.g., Yamashita and Jiang, 2010; Wolter and Yamashita, 2017; Ding and Reynolds, 2019; Fang and Zhang, 2021). These studies reported L1-L2 congruency effects by comparing the processing of congruent collocations to incongruent ones. The main findings are when there is an L1-L2 correspondence, processing is faster, and when there is no L1-L2 correspondence, processing tends to be disrupted (Yamashita and Jiang, 2010; Wolter and Gyllstad, 2011, 2013; Wolter and Yamashita, 2017; Sonbul and El-Dakhs, 2020). One account for these findings is that links to L1 collocations are utilized to map meanings of L2 collocations, thus leading to faster recognition of L2 collocations when there is an L1-L2 correspondence (Jiang, 2000; Yamashita and Jiang, 2010). Alternatively, Wolter and Yamashita (2017) explain that L2 learners are more likely to encounter L1-L2 collocations (i.e., since they are available in two sources of input) than incongruent items. They suggest that repeated exposure to congruent collocations leads to stronger entrenchment in memory for congruent collocations compared with incongruent collocations. This results in better and faster recognition of congruent items over incongruent items. A short representative summary of studies exploring congruency effects in collocations is provided in what follows.

Yamashita and Jiang (2010) explored congruency effects among Japanese learners of English. English Native speakers, English as a foreign language (EFL) learners, and English as a second language (ESL) learners were presented with congruent collocations (i.e., exist in both Japanese and English; e.g., *heavy stone*) and incongruent collocations (i.e., exist only in English; e.g., *kill time*), and were instructed to judge the phrases on whether they were acceptable in English. The response time and accuracy for English native speakers were the same in congruent and incongruent items. Japanese non-native speakers made more errors in incongruent items than congruent items. While Japanese EFL learners had longer response times in incongruent items than congruent items, Japanese ESL learners showed no difference in response times between the two item types. ESL learners were more advanced in proficiency than EFL learners, which led the authors to conclude that with increased L2 proficiency, the effect of L1 on processing fades away. However, many of the incongruent items had figurative meanings (e.g., *broken heart*). In contrast, most congruent items were transparent (e.g., *drink soup*). This resulted in congruency being confounded with figurativeness in the study.

Wolter and Gyllstad (2011, 2013) explored congruency effects on Swedish learners of English and had similar findings. In their earlier study, they used a lexical decision task in which learners were presented with the first word of the collocation and then were asked to judge whether the second word existed in English. English native speakers showed an identical advantage in processing both congruent and incongruent items. Non-native speakers showed an advantage in processing only congruent items. In the later study, they presented learners with congruent and incongruent phrases and instructed them to judge whether each phrase was common in English. The advantage of congruent items over incongruent ones was replicated in the second study. Notably, highly proficient learners were sensitive to frequency effects in the L2 but not the L1, regardless of whether an item was congruent. There was an effect of proficiency, such that higher proficiency learners recognized incongruent items with greater accuracy than lower proficiency learners. However, these two studies were limited. First, Wolter and Gyllstad (2011) did not use an objective estimate of L2 proficiency. Second, both studies used control items that were semantically implausible (e.g., *tell rug*). Furthermore, while the congruent items were transparent, some incongruent items in the two studies were opaque (e.g., *bite the bullet* in Wolter and Gyllstad, 2011; *tall order, dry spells* in Wolter and Gyllstad, 2013).

Similarly, Ding and Reynolds (2019) attempted to examine the link between congruency and proficiency. They compared performance on a phrase judgment task by Chinese learners of English with different proficiency levels and English native speakers. The results showed that the high proficiency group was more accurate on the congruent collocations and was faster in judging incongruent items than the low proficiency group. There was no difference between the two groups in response times for congruent items and in accuracy rate for incongruent items.

The effect of congruency on processing was also supported by Wolter and Yamashita (2017). They presented Japanese L2 learners with congruent (e.g., *strong wind*) and incongruent English-only (e.g., *low speed*) collocations in a phrase judgment task. Non-native speakers had faster processing of congruent over incongruent items, while native speakers showed equivalent performance in the two conditions. Notably, frequency interacted with proficiency, indicating that native speakers as well as higher proficiency learners were more sensitive to collocational frequency than lower proficiency learners. The authors suggested that with increased proficiency, learners become more sensitive to phrase frequency. However, the study had two limitations. First, control items were semantically implausible (e.g., *yellow society*), which may have limited the comparability between conditions. Second, incongruent items (e.g., *false teeth*) seemed less transparent than congruent items).

Pritchett et al. (2016) examined the effects of congruency on processing using a different set of items: English and Russian adjective-noun combinations of a figurative meaning. Russian English bilinguals were presented with the following items: English-only: *blue blood*; English-Russian: *blue moon*; Russian-only: *blue distances*. After the exposure phase, learners performed a recall test. Results showed that phrases that existed in the two languages were more easily recalled than phrases that existed in only one.

More recently, Fang and Zhang (2021) examined the combined effects of congruency, proficiency, and frequency on collocation processing. Congruent collocations (e.g., *black hole*) and incongruent collocations (e.g., *black tea*) were presented to English native speakers and Chinese learners of English in a phrase judgment task. Non-native speakers were more accurate in judging congruent than incongruent items, and higher proficiency learners were more accurate in judging incongruent items than lower proficiency learners. However, non-native speakers showed no processing advantage for congruent over incongruent items. The author explained that this may be because response accuracy and response time measured different things—explicit and implicit collocational knowledge, respectively. Surprisingly, native speakers were more accurate in congruent than incongruent collocations. This was attributed to the lack of control for transparency—congruent items were more transparent than incongruent items. Interestingly, the results indicated that L2 learners (i.e., especially those with lower proficiency) were more sensitive to word-level frequency than native speakers when processing collocations. The authors concluded that collocation processing is influenced by the combined effect of congruency, proficiency, frequency, and semantic transparency.

Sonbul and El-Dakhs (2020) examined the effects of congruency and proficiency on collocation processing. Sonbul and El-Dakhs employed both online (i.e., phrase judgment task) and offline measures (i.e., recognition task) to examine the recognition of congruent (e.g., *natural birth*) and incongruent collocations (e.g., *fresh start*) among Arabic L2 learners of English and English native speakers. In the recognition task, participants were given the noun of the collocation and were asked to choose the target collocation node among three distractors. The recognition task showed that both the congruency of the items and the proficiency of the learners were associated with greater accuracy. However, there was no interaction between congruency and proficiency, suggesting that the congruency effect was present across all proficiency levels. The online task revealed that native speakers had similar performance in accuracy and response times across the two types of collocations. Non-native speakers judged congruent collocations with better accuracy than incongruent ones. While non-native speakers had more correct responses in congruent than incongruent items, they had similar response times for both types of items. Proficiency modulated congruency effects for non-native speakers, such that the advantage for congruent over incongruent items in processing was evident for lower proficiency learners, but as proficiency increased, differences in response times based on congruency faded away, indicating a progression toward more native-like processing.

### The role of semantic transparency in collocation processing

In addition to congruency, semantic transparency is another potential factor affecting the processing collocations. Semantic transparency exists on a spectrum, where the line between a transparent and an opaque phrase is rather gradient (e.g., ranging from a very transparent item where the meaning of a collocation can easily be deduced from its parts as in *break his ankle*, to a less transparent one as in *break a record*, to a more opaque or idiomatic item where the meaning cannot easily be deduced from the parts as in *break the ice*). Laufer and Waldman (2011) explain that the figurative sense of a collocation (e.g., *face a problem*) is easier to comprehend than an idiom (e.g., *face the music*).

Although figurative collocations are widely used, they receive little attention in English teaching materials (Macis and Schmitt, 2016) and collocation research. In particular, many studies of collocations do not make a distinction based on the transparency of the items used. It is important to take semantic transparency into account since less transparent collocations cause more difficulty to L2 learners than more transparent ones (Macis and Schmitt, 2017).

Research on the effect of semantic transparency on processing has mostly focused on idiom processing. For example, Conklin and Schmitt (2008) compared the processing of idioms (e.g., *take the bull by the horns*) when embedded in a passage that supports either an idiomatic or a literal interpretation (“attack a problem” vs. “wrestle an animal”). The context in which the idiom was used (i.e., literal vs. idiomatic) yielded no difference in reading times between native and non-native speakers. This study came in contrast to Cieślicka's (2006) priming study, which examined the processing of idioms of multiple interpretations (e.g., *had cold feet*). Word targets associated with the literal meaning (e.g., *toes*) yielded larger priming effects than targets associated with the figurative meaning (e.g., *nervous*). Using eye-tracking, Siyanova-Chanturia et al. (2011) found that while native speakers showed similar reading patterns in literal and figurative contexts, non-native speakers read idioms that were used literally (e.g., *at the end of the day*: “in the evening”) faster than idioms that were used figuratively (e.g., *at the end of the day*: “eventually”).

While much research has examined the role of semantic transparency in idiom processing, very little research has explored its role in collocations. Gyllstad and Wolter (2016) examined the role of semantic transparency in the processing of collocations by asking participants to judge the meaningfulness of three types of items: free combinations (i.e., totally transparent items, e.g., *write a letter*), collocations (i.e., the adjective of the collocations is opaque, e.g., *run a risk*), and baseline non-collocational items (e.g., *carry a car*). They found that both native and non-native speakers had longer processing for the collocations compared to the free combinations. Both conditions were matched in phrase frequency, which led the authors to attribute the differences in processing to the semi-transparent nature of the collocations. More recently, Shi et al. (2023) conducted a self-paced reading experiment to investigate the effects of semantic transparency on collocation processing. Results showed that non-native speakers had longer processing for figurative collocations (e.g., *build a career*) compared to transparent ones (e.g., *build a house*). In contrast, native speakers showed no difference in performance across the two conditions.

### The present study

The review of the literature demonstrates that L1 knowledge facilitates L2 collocation processing (Yamashita and Jiang, 2010; Wolter and Gyllstad, 2011, 2013; Wolter and Yamashita, 2017). However, the review suggests two notable gaps in the literature.

First, despite several calls for more research on the effect of semantic transparency on the learning and processing of collocations (Webb et al., 2013; Gyllstad and Wolter, 2016; Pellicer-Sánchez, 2017), it has rarely been considered in previous research. Past research has either focused on collocations that were fully transparent or has not taken into account the varying degrees of transparency in the items selected. Thus, the picture is still unclear about the combined effects of semantic transparency and congruency on the processing of collocations, and it remains unclear whether the effects of congruency found in transparent collocations also extend to less transparent ones. Crucially, a large number of the items selected in previous research might be considered idioms (e.g., Yamashita and Jiang, 2010; Wolter and Gyllstad, 2011, 2013). The limitation of these studies is complicated by “the fact that the literal/figurative distinction is confounded with the congruent/incongruent classification” (Conklin and Carrol, 2019, p. 65). This suggests that incongruent items were more likely to be figurative than congruent items in previous research. Further, studies used semantically implausible word combinations as their control items (e.g., Wolter and Gyllstad, 2011, 2013; Wolter and Yamashita, 2017), which may have put control items at a disadvantage compared to target collocations.

Second, few studies have examined the relationship between congruency and proficiency (Yamashita and Jiang, 2010; Wolter and Gyllstad, 2013; Wolter and Yamashita, 2017), and these studies have revealed inconsistent findings about the relationship. Wolter and Gyllstad (2013) found an interaction between proficiency and congruency in accuracy but not response times, such that L2 proficiency improved accuracy rate, and this effect was larger for incongruent than congruent ones. On the other hand, Ding and Reynolds (2019) showed that the higher proficiency group was more accurate on congruent items and had faster recognition of incongruent items than their lower proficiency group. Yamashita and Jiang's (2010) study showed that only low-proficiency learners demonstrated a processing advantage based on congruency, indicating that congruency effects fade away with increased L2 proficiency. These studies suggest that the link between proficiency and congruency has not been established and that further research is needed to explore the relationship, which has pedagogical implications.

The present study aimed to address the above-mentioned limitations of previous research by further examining the relationship between congruency and the combined effects of proficiency and semantic transparency on the processing of collocations. The current study aimed to answer the following research questions:

1. How is the processing of L2 collocations influenced by (a) congruency, (b) L2 proficiency, and (c) semantic transparency?
2. Is the effect of congruency on L2 processing of collocations modulated by L2 proficiency and semantic transparency?

## Methods

### Participants

Arabic native speakers studying English as a foreign language (EFL) took part in the study (*n* = 106). All participants were 18–22-year-old undergraduate students studying English as a foreign language at a university in Saudi Arabia. They were at different levels of study, potentially reflecting different levels of English proficiency. Participants received course credit for their participation. The 1,000 (1 k) and 5,000 (5 k) levels of the Updated Vocabulary Levels Test (VLT), Version A (Webb et al., 2017) was used to assess participants' English proficiency. Participants were also asked to fill out a language background questionnaire that included questions about their age, their age when they first started to learn English, the period of time they stayed in an English-speaking country, and their estimates of their English proficiency in reading, writing, listening, speaking, and comprehension on a 7-point scale. Table 1 shows a summary of participants' proficiency and demographic data.

**Table 1:** Summary of data from the language background questionnaire.

	**Mean (SD)**
Age of first contact with the L2	10.25 (4.13)
Length of stay in an English-speaking country	7.80 (30.78)
VLT score (a 65-point scale)	47 (7.69)
Self-rating of Overall proficiency in English (a 7-point scale)	4.79 (1.16)
Self-rating of proficiency in speaking (a 7-point scale)	4.50 (1.28)
Self-rating of proficiency in understanding (a 7-point scale)	5.62 (1.16)
Self-rating of proficiency in reading (a 7-point scale)	5.37 (1.19)
Self-rating of proficiency in writing (a 7-point scale)	4.14 (1.36)

### Target items

Two categories of collocations were developed as stimuli (*n* = 24): (1) 12 incongruent English-only collocations that existed as collocations in English but not Arabic (e.g., *false teeth*), (2) 12 congruent English-Arabic collocations that existed in both English and Arabic (e.g., *fast food*). Collocations were either adjective-noun or verb-noun combinations, and this classification was applied equally across categories. Semantic transparency of the items was controlled for, such that half the collocations in each category were transparent (e.g., *fresh start*), and the other half were opaque (e.g., *false teeth*). The Corpus of Contemporary American English, COCA (Davies, 2008) was used to check the frequency of the collocations. Following Nguyen and Webb (2017), the phrase frequency of all collocations in COCA was >50, and the minimum score of mutual information was 3.0.

An additional 24 non-collocate items were used as control items to serve as a baseline for comparison. Each experimental item (i.e., congruent/incongruent) was matched with a control item that shared the same noun but had a different adjective/verb (e.g., for *reach an agreement*, the control item was *win an agreement*). There were two lists, such that the experimental item and its matched control item did not appear in the same list. None of the control items were actual collocations in English (mean phrase frequency in COCA = 2.47; range = 0.00–12 occurrences). A complete list of the stimuli used in the study is presented in Supplementary Table 1.

Congruency was operationalized in the study following Sonbul and El-Dakhs (2020). An English collocation was considered congruent if it had a direct literal translation in Arabic, which would also render it an acceptable collocation in Arabic. An item was considered incongruent if the literal translation of an English collocation into Arabic did not render an acceptable collocation in Arabic (i.e., there are no equivalent collocations in Arabic that matched the literal translation of *commit suicide* or *pay a visit*).

It was important to ensure that congruent and incongruent items were matched in their COCA frequencies. An analysis of variance with *post-hoc* Tukey comparisons between item types was conducted on log collocation frequencies. Results confirmed no significant differences in the collocation frequencies between incongruent items (*M* = 2.98, *SD* = 0.45) and congruent items (*M* = 3.10, *SD* = 0.57), *p* > 0.05, while control items (*M* = 0.36, *SD* = 0.37) were significantly lower than both experimental items, *p* < 0.05. An analysis of variance with *post-hoc* Tukey comparisons was also conducted on frequencies of the adjectives and nouns. There were no significant differences across the experimental item types for adjective and noun frequencies, *p* > 0.05. This suggests that experimental items were matched in both word and phrase frequency. Table 2 compares items in frequency, MI scores and length.

**Table 2:** Descriptive statistics of items (values in means).

	**Congruent items**	**Incongruent items**	**Control items**
Phrase frequency (log frequencies)	3.10	3.00	0.37
Frequency of the first word of the collocation (in log frequencies)	5.04	4.86	4.82
Frequency of the second word of the collocation (in log frequencies)	5.13	4.87	5.00
MI (Mutual Information)	6.07	7.00	–
Length of collocation (in letters)	12.48	11.70	12.82

### Measures

The role of congruency in online processing was assessed using a phrasal judgment task (PJT), a task that has been commonly used in the literature to gauge automatic recognition. In the task, participants were instructed to judge whether a word combination was commonly used in English. They were encouraged to respond as quickly and accurately as possible. The PJT was administered using PsychoPy 2 (Peirce et al., 2019), by which participants' accuracy and response latency were recorded. A response was coded as 1 (correct) or 0 (incorrect) based on participants' judgment of each item (collocation vs. control).

### Procedure

The study was carried out in accordance with the research ethics procedures at Imam Mohammad Ibn Saud Islamic University. The study took place in a quiet computer room in which participants were tested individually. Upon arrival, participants signed the consent form, which explained the study in general terms. Then, participants took part in the PJT. The exact instructions for the task, adapted from Wolter and Gyllstad (2013, p. 460), were as follows:

You need to decide whether the combinations presented are commonly used in English or not. Press the P button on the keyboard if the word combination is commonly used in English. Press the Q button on the keyboard if the word combination is NOT commonly used in English. For example, if you are presented with the phrase “strong wind,” please press P if you think this word combination is commonly used in English, and press Q if you think this word combination is not commonly used in English. Please answer as accurately and quickly as possible.

The task started with eight practice trials to familiarize participants with the task. Then, participants were presented with a total of 48 trials (24 collocate pairs and 24 control pairs), in which the order of presentation was randomized. Each trial was presented separately and was preceded by displaying a fixation target (series of 12 asterisks) in the middle of the monitor's screen for 1.000 ms, followed by a 50 ms blank screen. Immediately after that, the collocate pair was presented in the middle of the screen for 5,000 ms, or until participants made a decision.

At the end of the PJT, participants were asked to complete the two levels of the updated Vocabulary Levels Test (VLT; Webb et al., 2017), as well as the language background questionnaire. All tasks of the study were completed in one session, lasting ~30 min.

### Analysis

Initially, data were checked for outliers. Data were trimmed by removing response time values that fell above or below 2.5 standard deviations for each condition. Response times that were shorter than 500 ms were removed. This led to a loss of 0.86% of response time data.

The analysis was conducted using mixed-effects modeling in *R* version 3.6.1 (R Core Team, 2019). Accuracy data (correct = 1 or incorrect = 0) were analyzed by fitting logistic mixed-effects models using *glmer* function in the *lme4* package (Jaeger, 2008). Response time data (RT) were analyzed by fitting linear mixed-effects models using *lmer* function in the *lme4* package (Bates et al., 2014). Significance values were calculated using the lmerTest package (Kuznetsova et al., 2015), and interactions were examined using the *emmeans* package (Lenth, 2019).

The best-fit models for accuracy and RT were chosen based on likelihood ratio tests by including only predictors that contributed significantly to the final models. All models included random intercepts for subjects and items. Models included the following main predictors: Congruency (congruent or incongruent), Transparency (transparent or opaque), and Proficiency (VLT scores). The following covariates were also considered: Phrase Frequency (frequency of the collocation), Word Frequency (frequency of the first and second word of the collocation), Word Class (VN vs. AN collocations), MI (Mutual Information score), Length (number of letters in a phrase), and Trial number (to account for practice effects). RT data and all other continuous variables (e.g., frequency) were log-transformed to reduce skewness and to ensure that all variables were on the same scale.

## Results

Table 3 summarizes mean accuracy and RT across conditions. The first analysis compared response accuracies between experimental (congruent and incongruent) and control items. Results showed that experimental items (*M* = 74.52%, SD = 43.57) had significantly greater accuracy than their control non-collocate pairs (*M* = 56.40%, SD = 49.59), β = 0.94, *SE* = 0.065, z = 14.49, *p* < 0.001. Analysis of the control items was not considered further.

**Table 3:** Mean accuracy scores and RT (with standard deviations) in the PJT.

	**Accuracy score (%)**		**RT (ms)**	
**Type**	**Congruent**	**Incongruent**	**Congruent**	**Incongruent**
Transparent	89.62% (30.52)	66.66 % (47.20)	1,597 (700)	1,846 (889)
Opaque	76.36% (42.52)	65.06% (47.70)	1,598 (755)	1,702 (803)

Next, to evaluate the effectiveness of Congruency, the analysis only considered congruent and incongruent items. The best-fit mixed-effects logistic models, summarized in Table 4, showed that congruent items (*M* = 83.50%, SD = 37.14) yielded more correct responses than incongruent items (*M* = 65.56%, SD = 47.53). Phrase Frequency and W2 Frequency were significant factors, with higher frequencies of the phrase and word (i.e., the nouns) increasing the accuracy of responses. There was an interaction between Congruency and Proficiency. Analysis of the interaction indicated that Proficiency predicted accuracy of incongruent but not congruent responses, such that increased proficiency increased accuracy of responses for incongruent items. Transparency did not make a significant improvement in the model (χ^2^ = 2.03, *p* > 0.05).

**Table 4:** Model outcome for accuracy scores.

	**Accuracy**			
**Predictors**	**Log-odds**	**Std. error**	**Statistic**	***p***
(Intercept)	−5.94	2.48	−2.40	**0.017**
Condition [Incongruent]	−4.55	1.52	−3.00	**0.003**
Proficiency	0.23	0.38	0.59	0.552
Phrase frequency	0.39	0.15	2.56	**0.010**
log W2 frequency	0.38	0.18	2.09	**0.037**
Condition [Incongruent] × Proficiency	0.91	0.39	2.37	**0.018**
**Random effects**				
σ^2^	3.29			
τ_00__Subject_	0.51			
τ_00__Item Number_	1.12			

Bold values indicate statistical significance.

Analysis of RT data was carried out on the correct responses. A linear mixed-effects model was fitted to examine the effect of Congruency on RT. The model outputs showed that experimental items elicited shorter RTs (*M* = 1,663 ms, SD = 777) than their control non-collocate pairs (*M* = 2,284 ms, SD = 924), β = 0.23, SE = 0.01, *t* = −25.66, *p* < 0.001. Analysis of the control items was not considered further.

Next, the analysis of RTs only considered the experimental items. As shown in Table 5, congruent items (*M* = 1,597 ms, SD = 723) elicited faster RTs than incongruent items (*M* = 1,747 ms, SD = 833). Phrase Frequency was significant, such that higher collocational frequency predicted faster RTs. Trial number had a significant effect, with items eliciting shorter RTs as the experiment progressed. Neither Transparency (χ^2^ = 0.00, *p* > 0.05) nor Proficiency (χ^2^ = 2.43, *p* > 0.05) made a significant improvement in the model.

**Table 5:** Model outcome for RT.

	**RT**			
**Predictors**	**Estimates**	**Std. error**	**Statistic**	***p***
(Intercept)	7.84	0.13	59.50	**< 0.001**
Condition [Incongruent]	0.10	0.04	2.54	**0.011**
Phrase frequency	−0.06	0.02	−3.69	**< 0.001**
Trial number	−0.01	0.01	−1.99	**0.047**
**Random effects**				
σ^2^	0.10			
τ_00__Subject_	0.08			
τ_00__Item Number_	0.02			

Bold values indicate statistical significance.

## Discussion

Previous research has investigated the effect of congruency on the processing of collocations. However, previous research has either focused on transparent collocations or failed to control for transparency (e.g., *broken heart*), thus possibly confounding figurativeness with congruency. The present study aimed to investigate the combined effects of congruency, semantic transparency and proficiency on the processing of collocations. EFL participants (L1 = Arabic) were presented with English-only (e.g., *short notice*) and congruent collocations (e.g., *near future*) in a PJT, recording their RTs and judgment accuracy. The target items were controlled for transparency. Results of the present study will be discussed in relation to the wider literature.

The PJT showed that participants had a processing advantage for collocations compared to non-collocate control pairs. This suggests that non-native speakers exhibited frequency effects for collocations, as was demonstrated in previous research (e.g., Wolter and Gyllstad, 2013; Sonbul and El-Dakhs, 2020). This finding was also further supported in the patterns of results showing that learners were sensitive to phrase frequency: higher frequency collocations were associated with faster processing and greater judgment accuracy than lower frequency ones. The frequency effects for collocations can be explained in light of usage-based theories, which highlight the role of frequency in language learning and assume that frequency effects in processing extend single word *and* multiword sequences (e.g., Bybee, 1998, 2006; Tomasello, 2003; Goldberg, 2006).

The results showed that congruency had a facilitative effect on the processing of collocations. Congruent items yielded more correct responses and faster RTs than incongruent items. Learners exhibited congruency effects regardless of whether the items were transparent or opaque. The current findings support previous research demonstrating an advantage for congruent over incongruent collocations for L2 learners in response accuracy (Yamashita and Jiang, 2010; Wolter and Gyllstad, 2011, 2013; Ding and Reynolds, 2019; Sonbul and El-Dakhs, 2020; Fang and Zhang, 2021) and in RT (Wolter and Gyllstad, 2011, 2013; Wolter and Yamashita, 2015, 2017). However, a few other studies did not find an advantage in RT for congruent over incongruent collocations (e.g., Sonbul and El-Dakhs, 2020; Fang and Zhang, 2021). The absence of an L1 effect on RT in these studies might be due to the advanced L2 proficiency of their participants, with the effect of L1 diminishing with increased L2 proficiency (Sonbul and El-Dakhs, 2020).

The processing advantage for congruent collocations is likely due to the automatic activation of L1 translation equivalents (i.e., L1 collocational counterparts; Conklin and Carrol, 2019). The joint L1-L2 activation should facilitate the processing and judgment accuracy of congruent collocations. This view aligns with the revised hierarchical model (RHM; Kroll and Stewart, 1994), assuming a non-selective co-activation in processing across languages. An alternative explanation for the effect of congruency was made by Wolter and Yamashita (2017), who suggested that repeated exposure across more than one language provides more reinforcement for congruent collocations, resulting in further entrenching them in memory. As a result, the stronger entrenchment for congruent collocations leads to increasing familiarity, which accounts for their processing advantage.

In the current study, proficiency had an effect on processing, but it interacted with congruency: increased proficiency was associated with greater accuracy of responses for incongruent but not congruent items. This indicates that advanced L2 learners were more accurate on incongruent items than low-proficiency learners, supporting the findings of previous research (Yamashita and Jiang, 2010; Wolter and Gyllstad, 2013; Sonbul and El-Dakhs, 2020; Fang and Zhang, 2021). These patterns of results suggest that although congruency seems to influence collocation processing for both high and low-proficiency learners, its effects were more pronounced for low-proficiency learners. In other words, the effect of L1 on collocation processing was stronger for low-proficiency learners than high proficiency learners. Thus, it seems that effect of L1 on collocation processing starts to fade away gradually with increased L2 proficiency. There appears to be a progression toward more native-like processing with increased proficiency, potentially resulting in gradually diminishing the effects of L1 on L2 collocation processing. These findings lend support to RHM (Kroll and Stewart, 1994), suggesting that with increased L2 proficiency, a more direct route to the L2 lexicon becomes available, and the reliance on L1-mediation to access the meanings of L2 words diminishes.

The study demonstrated no evidence for an effect of transparency on collocation processing, whether in accuracy scores or in RTs. These results stand in contrast with Gyllstad and Wolter (2016), who found that opaque collocations exhibited slower processing than transparent ones. The discrepancy in results could be attributed to the number of items selected for each item type (opaque vs. transparent). There were 27 items for each item type in the Gyllstad and Wolter (2016) study, while there were only 12 items in the present study. Thus, the number of items in the current study might have been too small to provide the statistical power needed to detect an effect of transparency. Alternatively, another possible explanation for the lack of an effect of transparency in the present study is that opaque collocations were semi-transparent (e.g., *bitter experience*) and were not wholly non-compositional, which may have reduced the qualitative difference between transparent and opaque items. Finally, we cannot rule out the possibility that the lack of transparency effect might be because the transparency variable was treated dichotomously. Future research should consider these possibilities when investigating the influence of semantic transparency in collocation processing.

### Limitations and future directions

A number of important limitations need to be noted. First, the study did not recruit native speakers. Recruiting native speakers would provide a baseline for comparison and should further support the corpus-based (i.e., COCA) results that English-only and congruent items were matched in their frequencies. Second, transparency was treated dichotomously in this study, following Gyllstad and Wolter (2016). However, a dichotomous variable tends to reduce power (Baayen, 2010) and does not account for the variation in transparency within the items. Future research is thus suggested to identify transparency as a continuous variable by conducting norming studies that involve native speakers' assessment of the transparency of items. Further, it is likely that the number of items across the two transparency levels was too small to statistically detect an effect of transparency. Future replications should increase the number of items by treating transparency as a continuous variable, thus increasing statistical power. Finally, the task used in the study—the PJT– required participants to make an explicit judgment of the collocation. Although this task has been commonly used in previous studies on collocations, it does not directly tap into natural language processing. Other more sensitive psycholinguistic measures, such as eye-tracking, allow for a more natural form of reading and thus provide a more direct measure of language processing. However, little research has utilized eye-tracking to assess the role of congruency in the processing of collocations. Further research is thus needed to explore congruency effects using methods like eye-tracking to advance our understanding of how congruency influences collocation processing.

## Conclusion

The present study contributes to our understanding of formulaic language processing by investigating the role of congruency in the processing of collocations while controlling for transparency. This factor has not been previously considered in previous research. The study demonstrates the effect of congruency on collocation processing: non-native speakers had a processing advantage for congruent collocations compared to incongruent ones, reflected in their RTs and accuracy. Differences between congruent and incongruent items seem to fade away with increased proficiency, suggesting a shift toward more native-like processing with increased proficiency. These findings have an important implication for L2 teaching: incongruent formulaic sequences should receive more attention than congruent ones for low-proficiency learners since the learning of congruent items is likely to be facilitated. Results showed that transparency had no effect, with no difference in RTs and accuracy between transparent and opaque collocations. The small number of items selected and the fact that transparency was treated dichotomously might account for the lack of transparency effect on the study. This highlights the need for further research to better understand the role of transparency in collocation processing.

## Data Availability

The datasets presented in this study can be found in online repositories. The names of the repository/repositories and accession number(s) can be found at: https://osf.io/7wgqc/?view_only=a644834978744d9fb20815d0477e54dd.
