# Supplementary material for: The effects of cross-language overlap and semantic transparency on the processing of L2 collocations
Source: Front Psychol. 2024 Aug 29;15:1417786. doi: 10.3389/fpsyg.2024.1417786 (PMC11391528; doi:10.3389/fpsyg.2024.1417786)
Supplement: Supplementary file 1 [file Table_1.docx]

| **Table S1. List of the Collocations Used in the Study** | | | |
| --- | --- | --- | --- |
| **Item** | **Congruency type** | **Transparency Type** | **Control pair** |
| collect information | Congruent | Transparent | achieve information |
| comfortable chair | Congruent | Transparent | amazing chair |
| use force | Congruent | Transparent | prepare force |
| follow the instructions | Congruent | Transparent | keep the instructions |
| near future | Congruent | Transparent | available future |
| side effects | Congruent | Transparent | tiny effects |
| bitter experience | Congruent | Opaque | keen experience |
| fall victim | Congruent | Opaque | drop victim |
| fast food | Congruent | Opaque | weak food |
| green light | Congruent | Opaque | extensive light |
| improve the image | Congruent | Opaque | assist the image |
| play a role | Congruent | Opaque | deliver a role |
| bridge the gap | Incongruent | Transparent | limit the gap |
| commit suicide | Incongruent | Transparent | apply suicide |
| fresh start | Incongruent | Transparent | current start |
| give birth | Incongruent | Transparent | present birth |
| grand jury | Incongruent | Transparent | executive jury |
| late afternoon | Incongruent | Transparent | past afternoon |
| catch a cold | Incongruent | Opaque | attract a cold |
| common sense | Incongruent | Opaque | average sense |
| lend a hand | Incongruent | Opaque | send a hand |
| false teeth | Incongruent | Opaque | pure teeth |
| meet challenges | Incongruent | Opaque | treat challenges |
| naked eye | Incongruent | Opaque | unique eye |
| waste time | Congruent | Transparent | ruin time |
| reach an agreement | Congruent | Transparent | win an agreement |
| solve a problem | Congruent | Transparent | judge a problem |
| lose money | Congruent | Transparent | cancel money |
| last chance | Congruent | Transparent | basic chance |
| accept the fact | Congruent | Transparent | pick the fact |
| strong coffee | Congruent | Opaque | hard coffee |
| blind obedience | Congruent | Opaque | wide obedience |
| high cost | Congruent | Opaque | fine cost |
| put an end | Congruent | Opaque | show an end |
| golden opportunity | Congruent | Opaque | original opportunity |
| black market | Congruent | Opaque | criminal market |
| short notice | Incongruent | Transparent | terrible notice |
| pay a visit | Incongruent | Opaque | offer a visit |
| plastic surgery | Incongruent | Opaque | artificial surgery |
| poor performance | Incongruent | Opaque | wrong performance |
| soft drinks | Incongruent | Opaque | easy drinks |
| throw a party | Incongruent | Opaque | receive a party |
| hit the road | Incongruent | Opaque | quit the road |
| break the law | Incongruent | Opaque | skip the law |
| draw a conclusion | Incongruent | Opaque | hold a conclusion |
| carry the burden | Incongruent | Opaque | move the burden |
| heavy traffic | Incongruent | Opaque | difficult traffic |
| honest mistake | Incongruent | Opaque | regular mistake |
